# Bodily Experience Among Arab Therapists in Israel and Its Expressions in Psychotherapy

**DOI:** 10.1007/s11013-026-10024-1

**Published:** 2026-11-13

**Authors:** Hanan Knani, Shai Shorer

**Affiliations:** Faculty of Social welfare and Health, School of Social Work, University of Haifa, Haifa, Israel

**Keywords:** Psychotherapy, Embodiment, Somatic countertransference, Culture, Arab female therapists

## Abstract

Embodied experience is increasingly recognized as central to psychotherapy, yet relatively little is known about how therapists experience and make sense of bodily phenomena in clinical practice, particularly non-Western cultural contexts. This study explored how Israeli Arab female psychotherapists experience, interpret, and engage with their bodies during therapeutic encounters, including how these experiences are shaped by the intersections of culture, gender, professional training, and lived experience. Semistructured in-depth interviews were conducted with 20 Arab female therapists and analyzed using interpretative phenomenological analysis. Participants described the body as a source of clinical knowledge, emotional attunement, and therapeutic understanding. Three interrelated themes emerged: the body as a culturally attuned therapeutic medium; the therapist’s bodily journey between traditional culture and professional development; and the use of embodied knowledge to challenge professional and cultural boundaries. Findings suggest that embodied awareness is not merely a professional competency acquired through formal training, but also an evolving process shaped through personal experience, clinical practice, cultural meanings, and professional development. This study contributes to a culturally informed understanding of embodiment in psychotherapy by demonstrating how in some cultural contexts where emotional experiences are typically restricted, bodily sensations can be an important channel for emotional communication and clinical knowledge. In such circumstances, therapists often continuously negotiate between traditional cultural expectations and psychotherapeutic knowledge, using their embodied experiences to navigate tensions among personal, cultural, and professional worlds. Hence, embodiment is not only a therapeutic resource but also a culturally shaped process through which professional practice is developed and transformed. The findings emphasize the importance of addressing embodiment, culture, and gender more explicitly in psychotherapeutic training and clinical practice.

## Introduction

### Embodiment, Culture, and Therapeutic Practice

Cultural norms, gendered expectations, social values, and power relations shape how bodies are experienced, regulated, interpreted, and made present in everyday life (Holmqvist Gattario et al., 2020; Ussher, 2022). Therefore, embodied experience cannot be understood as a universal or purely individual phenomenon, but rather as one that emerges through the sociocultural contexts in which people live. The meanings assigned to bodily sensations, emotions, and forms of self-expression are shaped by broader cultural and historical processes, influencing how individuals understand themselves and relate to others (Ussher, 2022). These influences affect not only clients but also therapists, whose embodied experiences are inseparable from the social and cultural worlds they inhabit and the relational contexts in which their therapeutic work unfolds (Raval et al., 2024). Attending to therapists’ embodied experiences, therefore, may offer a unique lens through which to examine the intersections of culture, gender, and professional practice in psychotherapy.

### The Place of the Body in Contemporary Psychotherapy

Despite the fundamental role of embodied experience in human life, the body has often occupied an ambiguous position in Western psychological thought. Historians of psychiatry and psychology have noted that Cartesian dualism has profoundly influenced modern understandings of mind and body, conceptualizing them as distinct entities and prompting debates in psychology, psychiatry, and neuroscience (Berrios, 2018). Hence, in many psychological traditions, emotional life and psychological suffering consequently have been conceptualized primarily through cognitive, symbolic, and verbal processes, whereas bodily experience has often remained in the background. Recent writings have called for greater integration of embodied processes into psychotherapy, criticizing reductionist and exclusively brain-based approaches and highlighting the therapeutic potential of embodiment for emotional regulation, self-awareness, and psychological change (Fugate et al., 2024).

Rather than viewing the body as a passive container of psychological processes, embodiment perspectives conceptualize bodily experience as fundamental to perception, emotion, meaning-making, and interpersonal understanding (Fugate et al., 2024). In psychotherapy, embodied approaches emphasize that bodily sensations, movement, posture, and physiological responses can provide important sources of clinical information and contribute to therapeutic change (Ogden & Fisher, 2015). Accordingly, therapists are understood not as detached observers but as embodied participants whose bodily experiences contribute to interrelational attunement, understanding, and the therapeutic relationship.

Embodied experience is shaped by not only psychological processes but also gendered social and cultural conditions, such that social norms, power relations, and culturally prescribed expectations regarding femininity and bodily conduct play a major role (Holmqvist Gattario et al., 2020). Feminist scholars have further argued that women’s bodies cannot be understood solely through biological processes, because bodily experiences are continuously shaped by social, cultural, and political factors. Importantly, they have highlighted how women’s bodies are shaped through processes of regulation and pathologization across diverse cultural and historical contexts (Ussher, 2022). Western psychological, psychiatric, and medical traditions have historically contributed to defining norms of femininity, shaping restrictive assumptions about women’s bodies and emotions and regulating the meanings attached to their bodily experiences (Ussher, 2022). Women’s embodied experiences, therefore, emerge through ongoing negotiations among bodily realities, cultural meanings, gender norms, and social power relations (Holmqvist Gattario et al., 2020; Ussher, 2022).

### Psychotherapy and Embodiment in Arab Society

Arab society in Israel is shaped by multiple cultural, familial, religious, and sociopolitical systems of meaning (Abu-Baker, 2016). In this context, therapeutic encounters unfold at the intersection of diverse, and at times contradicting, value systems and understandings of selfhood, relationships, and healing. Although many psychotherapeutic models are grounded in assumptions that emerged in individualistic Western societies, Arab communities are often characterized by more collectivistic orientations in which personhood is understood in relation to family, community, and social obligations (Dwairy, 2023). As a result, tensions may arise when psychotherapeutic models encourage individual self-exploration and the expression of personally meaningful wishes and emotions, whereas traditional cultural frameworks emphasize social harmony, family loyalty, and adaptation to collective expectations (Dwairy, 2023).

Arab clients and therapists, therefore, frequently navigate between therapeutic frameworks rooted in Western psychology and local understandings of emotional experience, interpersonal relationships, and healing (Daass-Iraqi et al., 2021). These intersecting frameworks shape not only help-seeking practices but also the meanings attributed to emotions, bodily experiences, and interpersonal relationships (Daass-Iraqi et al., 2021). Consequently, culturally responsive psychotherapy requires attention to the social and cultural contexts in which distress is experienced and expressed, including the roles of family, gender, religion, and community (Daass-Iraqi et al., 2021; Dwairy, 2023).

Previous research has highlighted the importance of culturally responsive psychotherapy in Arab society and other collectivistic cultural contexts, emphasizing the need to adapt therapeutic interventions to local systems of meaning, family structures, religious beliefs, and community networks, rather than relying exclusively on assumptions derived from Western individualistic models (Al-Krenawi & Graham, 1999; Daass-Iraqi et al., 2021; Dwairy, 2023; Ennis et al., 2020). Studies of Arab therapists in Israel have documented the challenges involved in reconciling Western professional training and the cultural norms of Arab society, often requiring therapists to negotiate tensions between professional principles and local realities and leading them to develop hybrid professional identities and culturally adaptive practices (Bar Zaken & Walsh, 2021).

### Research Gaps

Despite the growing literature on cultural adaptation and culturally responsive psychotherapy, relatively little is known about how Arab female therapists experience and use their bodily knowledge in psychotherapy (Al-Krenawi & Graham, 1999; Bar Zaken & Walsh, 2021; Daass-Iraqi et al., 2021) and how their embodied experiences shape clinical practice. Because therapists’ professional development and clinical practice are shaped through not only formal training but also personal experiences, clinical encounters, and context-dependent learning processes (Raval et al., 2024), it is crucial to investigate these encounters in various cultural contexts. Emerging research has suggested that practitioners’ lived experiences may become intertwined with their professional identities and influence how they understand and engage in therapeutic work (Stjernegaard et al., 2025). However, little research has examined these processes among Arab women therapists in Israel or explored how their embodied experiences are negotiated at the intersections of gender, culture, professional identity, and sociopolitical context. The current study aimed to help fill this gap.

### Study Aims

This study explored how Arab women therapists in Israel experience and make sense of bodily phenomena in psychotherapy. To address this question, this research drew on in-depth interviews with 20 Arab women therapists from diverse professional backgrounds working across public, private, educational, and healthcare settings. It examined how embodied experiences are understood, interpreted, and integrated into therapeutic work. Particular attention was given to how therapists’ bodily experiences are shaped by the intersections of culture, gender, professional training, and lived experience. By focusing on therapists’ embodied perspectives, it aimed to contribute to the growing literature on embodiment in psychotherapy by highlighting how the therapist’s body may serve as not only a source of clinical information but also a site through which personal histories, cultural meanings, and professional knowledge are negotiated and transformed.

The study addressed the following questions:

1. How do Arab women psychotherapists perceive their bodily experience in therapy and its impact on the course of treatment?
2. What are the salient characteristics of therapists’ bodily experience during psychotherapy, and how does this experience manifest in the therapeutic encounter?
3. In what ways does attention to bodily dimensions in therapy support or hinder cultural responsiveness in psychotherapy with Arab clients in Israel?

### Method

This study employed an interpretive phenomenological approach (IPA) to explore how Arab women therapists in Israel make sense of embodied experiences in psychotherapy. IPA is concerned with how individuals interpret and attribute meaning to lived experience and is particularly suited to examining experiences situated in specific cultural and relational contexts (Smith et al., 2022). Consistent with this approach, the study focused on how bodily experiences are understood, interpreted, and integrated into therapeutic practice in the sociocultural context of Arab society in Israel.

### Sampling and Participants

Participants were recruited through criterion sampling. All interviewees were Israeli Arab trained mental health professionals from paramedical therapeutic fields. To be eligible, participants needed at least a bachelor’s degree, with preference given to therapists holding a master’s degree in social work, psychology, or creative arts therapy.

The study focused on Arab therapists who provide emotional therapy in public and private settings and work primarily with Arab clients. Recruitment began with announcements in professional social media groups inviting therapists to inquire about participation by phone or email. Subsequently, snowball sampling (Heckathorn & Cameron, 2017) was used: Participants who agreed to participate then referred additional therapists, providing them with information about the study and sharing the research team’s contact details.

Recruitment continued until sufficient depth and richness of experiential accounts had been obtained to support a nuanced interpretative analysis of participants’ embodied experiences (Smith et al., 2022). Accordingly, 20 Arab women therapists participated in the study. Participants’ demographic and professional characteristics are presented in Table 1. On average, they were 39.4 (*SD* = 4.2) years old and had 15.2 (*SD* = 5.6) years of clinical experience.

**Table 1:** Participants’ demographic and professional characteristics

Pseudonym	Age	Profession	Years of clinical experience	Current work setting
Miriam	46	Clinical social worker, PhD	24	Private practice
Balqis	47	Clinical social worker MSW	25	Private practice, public social services
Shahd	48	Movement therapist, MA	11	Hospital
Moheba	40	Drama and art therapist, PhD	18	Educational setting, private practice
Ru’ya	39	Movement therapist, MA	13	Hospital, private practice, public social services
Samah	34	Dance and movement therapist, MA	11	Educational setting
Salima	29	Psychologist, MA	5	Healthcare services
Tala	40	Clinical social worker, MSW	18	Healthcare services
Alin	36	Psychologist, MA	12	Healthcare services
Rimas	28	Psychologist, MA	4	Healthcare services
Sham	42	Family therapist, MA	17	Public social services
Jana	45	Psychologist, MA	21	Healthcare services
Talin	49	Clinical social worker, MSW	24	Healthcare services
Somaya	25	Psychologist, BA	2	Hospital
Ahlam	38	Educational psychologist, MA	15	Healthcare services
Riham	40	Clinical social worker, MSW	15	Healthcare services
Zainab	40	Clinical social worker, MSW	17	Healthcare services
Afaf	36	Art therapist, MA	13	Public social services
Zina	46	Clinical social worker, MSW	24	Public social services
Tajreed	40	Art and energy-healing therapist, MA	16	Educational setting, private practice

### Procedure and Data Collection

The study was conducted in accordance with the approval of the authors’ Faculty Ethics Committee (#179/25). Data were collected through semistructured in-depth interviews, which allowed exploration of participants’ subjective experiences and the meanings they attribute to them (Kvale & Brinkmann, 2009).

An interview guide was developed based on the literature review and the study’s central research questions. It served as a flexible map rather than a rigid structure, allowing the conversation to develop organically and enabling deeper exploration of themes that arose spontaneously during the interviews (Brinkmann, 2016).

The participants selected the interview location. Interviews were conducted face to face and lasted approximately 90 minutes each. Interviews began with broad, open-ended questions aimed at eliciting the therapist’s general therapeutic experience, such as:

- Please describe two to three central characteristics of your therapeutic work.
- What are the primary challenges you encounter with clients?

Subsequent questions focused on embodied experience, such as:

- How is bodily experience addressed in your therapeutic work?
- How do you experience your body during sessions?
- How, if at all, does this vary across clients?

All interviews were conducted by the first author in the participants’ preferred language (Arabic), audio recorded, transcribed verbatim, and translated to Hebrew for supervision purposes by the first author. Selected excerpts were translated into English for inclusion in the present article.

### Data Analysis

Data analysis was conducted in accordance with the principles of IPA, which aims to explore how individuals make sense of their lived experiences through detailed, idiographic, and interpretative engagement with their accounts (Smith et al., 2022). Analysis proceeded through an iterative, multistage process. First, each transcript was read several times to achieve close familiarity with the participant’s narrative. Initial notes were made, focusing on descriptive content, linguistic features, and preliminary interpretive comments. These notes were then developed into emergent experiential themes for each individual. Following this idiographic analysis, patterns of convergence and divergence were examined across participants, allowing for the identification of shared themes while preserving individual nuance and specificity.

Throughout the analytic process, attention was given to the participants’ meaning-making and the double hermeneutic central to IPA, whereby the researcher seeks to make sense of participants who are similarly engaged in making sense of their experiences (Smith, 2007). The analysis involved continual movement between parts and whole and between individual narratives and the developing thematic structure to ensure depth, coherence, and grounding in the data (Smith et al., 2022).

### Methodological Integrity

In line with contemporary approaches to methodological integrity in qualitative research (e.g., Levitt et al., 2017), reflexive, transparent, and iterative research practices were employed to support coherence among the study’s aims, methods, and analytic procedures. The primary researcher maintained a reflexive journal throughout the study, documenting methodological decisions, analytic reflections, and considerations related to her positionality as an Arab woman therapist and researcher. Ongoing discussions with the academic supervisor and coauthor, who is an experienced scholar in the field of cross-cultural psychology and has familiarity with the study context, supported critical reflection on emerging interpretations and analytic decisions. Analysis involved repeated engagement with the interview transcripts and continual movement between participants’ accounts and developing interpretations. Interpretations were grounded in participants’ narratives, and verbatim quotations were used to illustrate analytic claims. Systematic documentation of recruitment, data collection, and analytic procedures further enhanced transparency and coherence throughout the study.

### Ethical Considerations

As previously noted, this study received approval from an institutional ethics committee. Oral informed consent was obtained from all participants and recorded at the start of each interview. Participants were assured of their confidentiality and informed that data would be used solely for this study.

Given the personal and potentially distressing nature of the study topic, interviews were conducted with sensitivity and attunement to signs of discomfort. Participants had the option to pause or discontinue the interview at any stage. A list of professional support resources was provided for cases of emotional difficulty. However, none of the participants chose to seek further support.

All recordings and transcripts were stored securely, accessible only to the primary researcher. Identifiable information was removed during transcription and replaced with pseudonyms and participant codes. Any identifying details were modified or omitted to preserve anonymity.

## Findings

The analysis yielded three main themes. The first, “The body as a culturally attuned therapeutic medium,” presents the body as a source of knowledge and arena in which emotional expression and containment occur during therapeutic encounters. The second, “The therapist’s bodily journey: between traditional culture and professional development,” deals with the process of Arab female therapists who learn to use the body as a professional tool and how this shapes their professional identity. Finally, the third theme, “From the clinic to research: using embodied knowledge to challenge professional and cultural boundaries,” describes how Arab female therapists use embodied and cultural knowledge to challenge the boundaries of professional and cultural discourse in their workplaces.

### The Body as a Culturally Attuned Therapeutic Medium

Most participants described their body as a “listening instrument” that provides nonverbal emotional knowledge about both the client’s inner world and their own. They described bodily experience as preceding words and emerging through sensations such as breathing, movement, or internal states, shaping the therapeutic dialog. Attending collaboratively to these sensations helped deepen the therapeutic relationship. Samah (34, dance and movement therapist) explained:

Sometimes when a client tells me about chest pain, I also feel this kind of pressure. … I ask about it: “I feel this and that. Do you feel it, too?” I put my hand on my chest and check—is it mine, hers, or ours?

Her description illustrates how bodily awareness can become a shared sensory channel that enables emotional expression when verbal language is limited, while remaining aligned with cultural norms of modesty and restraint.

Several participants highlighted the centrality of the body in emotional expression in Arab society. Ru’ya (39, dance and movement therapist) described how bodily language often is a practical entry point for emotional exploration when clients have limited familiarity with naming feelings:

If you sit and talk with a [Jewish] client about where she feels the pain and what it does to her—where she feels it in the body, the fear, for example—she answers right away. … “I feel it here. Something in my legs.” … With [Bedouin] clients, I almost always use introductions to explain what this emotion is and where they felt it, … because when I say, “You feel disappointed,” they ask, “What is disappointment?” It’s like teaching a new language.

This excerpt highlights how the body functions as an anchor for emotional inquiry when a shared emotional vocabulary cannot be assumed. Bodily sensation serves as a scaffold for identifying emotions, often requiring brief preparatory framing before or alongside naming feelings.

Several participants emphasized that bodily metaphors were more culturally acceptable than naming emotions directly. For example, Rimas (28, psychologist) recalled a client who expressed distress by saying: “They broke my back.” Similarly, she recounted a bereaved mother who referred to her distress as “seeing darkness in the room,” rather than naming fear or anxiety directly. In a similar vein, Tala (40, clinical social worker) described a woman who articulated her sense of helplessness by saying, “There is nothing in my hands,” using a bodily metaphor to convey powerlessness and lack of agency.

Religious and spiritual dimensions were also intertwined with bodily experience in therapy, appearing through beliefs in a higher power, energy work, prayers, angels, and traditional rituals. Participants described the body as a gateway to spiritual presence that provided protection and strength. Tajreed (40, art and energy-healing therapist), who integrates body-oriented energy work with touch-based practices, described the importance of embodied presence and structured rituals of protection:

I feel enough strength that I can serve as the container and hold everything that comes up. … And of course, in energy work—especially any work that combines body and psyche—I rely a lot on angels and their protection. … At the beginning, I always ask [Archangel Michael] to wrap me in a kind of circle, like a bubble that protects me, … and there’s also a way to close the magnetic field between you and her at the end of the treatment.

This account frames spiritual protection as an embodied clinical technique through which Tajreed positions herself as a container and maintains boundaries. Practices such as “wrapping,” creating a protective “bubble,” and closing a “magnetic field” enact bodily and spiritual containment, allowing her to remain present without carrying the client’s experiences beyond the session.

Other participants described how clients’ religious considerations shape what was permissible to explore in therapy. Miriam (46, clinical social worker) explained: “I remember one orthodox Muslim client who would excessively talk about religion. … Everything I suggested, … she checked whether it was acceptable according to religion.” Such reliance on religious approval influenced the pace and depth of therapy and required careful adaptation of interventions.

Some participants described the body as a site where defense mechanisms became visible in therapy. Riham (41, clinical social worker) described a moment of bodily paralysis and intrusion: “The client wanted to know more about me. … There was something threatening about him. I felt frozen and couldn’t breathe. … The paralysis was physical, and also at the level of my thinking.”

Riham’s account illustrates how bodily paralysis functioned as an immediate signal of threat, disrupting both physical presence and clinical thinking. Similarly, Salima (29), a psychologist working in a day-treatment hospital and an outpatient clinic serving predominantly Arab clients, described how embodied sensations guided her clinical decisions in encounters experienced as unsafe:

At some point, I realized I had to stop the therapy. Physically and mentally, it felt invasive and threatening. … I noticed rapid heartbeats and sometimes nausea. … This experience reminded me of an earlier situation in a Bedouin village, where a man harassed me. … In both cases, it felt like my “no” was not being heard.

Salima’s account illustrates how bodily distress functioned as an early warning system signaling a breakdown of safety in the therapeutic relationship. Her somatic reactions were embedded in a broader gendered and sociocultural context, guiding her toward withdrawal as an act of self-protection and ethical boundary-setting.

Whereas Salima’s narrative emphasized acute bodily alarm, other participants described how prolonged emotional responsibility accumulated more gradually in the body. Talin (49, clinical social worker) reflected: “When I was managing the treatment center, I suffered from terrible pain in my lower back. … Once I understood this, … the pain was released.” Her account reflects a culturally resonant understanding in which emotional responsibility and professional dedication are carried in the body until they are consciously acknowledged.

In sum, participants experienced their bodies as sensitive, meaning-laden instruments through which emotional, cultural, and spiritual knowledge was sensed and negotiated in therapy. Bodily attunement functioned as a core professional tool for understanding of clients, self-regulation, and maintenance of culturally attuned therapeutic boundaries.

### The Therapist’s Bodily Journey: Between Traditional Culture and Professional Development

Most participants described beginning their professional paths with experiences of bodily restraint and limited permission to attend to or speak about the body, shaped by social and familial norms. Over time, through professional training and clinical practice, they gradually opened to learning through embodied experience. This process enabled the emergence of a professional identity that mediated traditional cultural norms and Western psychotherapeutic approaches.

Many participants grew up in cultural contexts in which the female body was constantly supervised and criticized, and speaking about the body was associated with shame, restrictions on touch, and parental control. Moheba (40, drama therapist and arts-based psychotherapist) described a sustained effort to translate Western clinical training into culturally resonant practice, emphasizing that embodied and action-based work is central to her modality, yet often culturally unfamiliar:

Very few women are connected to their bodies. For us, women are used to feeling as if their bodies are distant machines. … I think we Arab women have an issue with the body. In adolescence, I also understood that there are body areas that carry a particular kind of shame.

Moheba’s words point to a culturally learned disconnection from the body, in which embodied awareness is restricted rather than cultivated. In this context, embodied therapeutic work involves gradual, carefully paced invitations that renegotiate cultural meanings attached to the female body.

For many participants, leaving their hometowns for higher education marked their first legitimate access to bodily experience. They perceived therapeutic training as culturally novel. Zainab (40, clinical social worker) described her studies as the first context in which bodily awareness became accessible to her: “In my studies, I learned how to breathe, to notice and identify different sensations, … as if I was allowed to feel myself. It was a lesson for life, not just academic learning.” Her words reflect not only longstanding distance from the body, but also the experience of receiving cultural permission to inhabit her body.

Others described professional training as a catalyst for conceptual and personal change. Balqis (47, clinical social worker) described her training as a turning point in how she relates to her body:

The studies confirmed something that was already inside me. … Today, I’m more aware of my body. I understand that pain is not always medical. I see the body as part of the psyche. As for myself, too, it’s a process. I’m learning to stop, to listen, to notice when my body signals and when I need to recharge.

Her account illustrates a gradual shift from perceiving the body as primarily functional or medical toward experiencing it as a source of emotional knowledge and self-regulation. This ongoing process reflects the development of a professional identity in which bodily awareness becomes both a personal resource and a clinical tool, cultivated through training rather than assumed in her cultural upbringing.

Encountering embodied therapeutic approaches placed participants between two value systems—one emphasizing restraint and control, and the other openness and inquiry. They often experienced this juxtaposition as both a “culture shock” and a portal to growth. Tajreed (40, art and energy-healing therapist) described this shift:

I don’t want to continue living according to prohibitions that were imposed on me. I need to check why this is forbidden and whether it fits me or not. First, I observed my body’s reactions to the things I learned—where it feels comfortable and where not. … I understood that I need to experience and believe in it in the deepest cell of my body before I suggest this way of healing to others.

For Tajreed, embodied awareness became a compass for self-understanding and a foundation for therapeutic work.

Many therapists described an ongoing process of translating Western academic knowledge into culturally resonant therapeutic language, learning to “start from the familiar” to build safety and trust. Ru’ya (39, dance and movement therapist) described the gap she initially encountered between theory and practice:

There is a gap between theoretical studies and the actual field. … I went through a very long process until I clarified the mind–body connection for myself. … And when I come to a client who doesn’t have this background … and start ask him to express himself through movement, there is usually a lack of understanding.

Ru’ya described this gap based on her early professional uncertainty, which led her to seek creative cultural bridges. Incorporating traditional folk dance (Debka) allowed her to work through culturally familiar forms of movement and emotional expression, translating academic language into community-based practice.

Although embodied training expanded therapists’ sense of agency, some participants emphasized learning to recognize bodily limits as part of their professional maturation. Jana (45, psychologist) reflected on how bodily vulnerability shaped her therapeutic stance:

I live with a chronic illness, and in that sense, my body constantly speaks to me. I learned to live with it rather than recover fully, and perhaps because of this, I am more attentive to bodily signals—fatigue, pain, anxiety. There are moments when I ask myself whether to hold sessions or to rest, because my body is present in the therapeutic space. At the same time, I remind myself that the client comes to receive care, not to take care of me.

Jana’s account highlights how bodily awareness can emerge through lived vulnerability and professional training. Her illness sensitized her to somatic cues while sharpening ethical boundaries, positioning the body as both a source of clinical attunement and a site requiring careful regulation.

Taken together, participants’ narratives reveal a therapeutic identity rooted in place, body, and culture, depicting a movement from bodies shaped by social restriction to bodies capable of attunement, expression, and professional agency. Embodied awareness became both a personal evolution and a clinical tool, shaping a therapeutic identity grounded in cultural translation and ethical responsibility.

### From the Clinic to Research: Using Embodied Knowledge to Challenge Professional and Cultural Boundaries

Most participants described academic, professional, and experiential learning processes in which they were required to bridge the worlds they inhabit: academic institutions, Western knowledge, and local cultural wisdom. Samah (34, dance and movement therapist) articulated this tension:

In our society and in Islam, there is a belief in body–mind and the soul, which correspond to the higher self and lower self in mental health concepts. On the other hand, there were big differences between academia and the field. I learned Western culturally based theories and then had to adapt them to my clients.

Many participants pointed to a gap between the academic knowledge they acquired and the social and cultural reality in which they work. They experienced academic training as offering a Western therapeutic language that sometimes felt too narrow and distant from the lived experiences of their Arab clients. This gap, however, became a driving force for creating a new therapeutic language. Moheba (40, art therapist) described this concept:

In the first years, I really wanted to take everything I learned and apply it to my community, but something didn’t work. I began to realize that most psychology is Western and not adapted to our context. For example, parents would bring their son to “fix” him—to make him obedient, because hierarchy is so central in our society. And it wasn’t only psychology. Even the art we studied was often not adapted to Arab culture. We were taught Greek mythology; how am I supposed to tell an Arab woman about Medea or someone like that, when it doesn’t connect to her existing language? So, I started looking for things closer to our world: our heritage, our dances, our movements, even our roles in the family and society. They have a particular composition, and it’s different. That was, and still is, a major challenge.

Moheba’s account illustrates how academic training offered a therapeutic language that often felt culturally distant from the lived realities of Arab citizens of Israel. She experienced this gap in both clinical expectations (e.g., hierarchy and obedience) and the symbolic material available for meaning-making in therapy. Her search for heritage-based movement and culturally resonant references marks an early step in creating a hybrid, embodied therapeutic language.

Other therapists described how academic and professional training set in motion a personal journey of inquiry, reflection, and boundary expansion. Moheba said:

We were never taught this process. No one came and said: “Think about culturally sensitive psychology.” I think I began to understand it only later—and honestly, my turn toward doctoral studies came out of real distress, because something wasn’t working for me. It didn’t sit right.

There is a huge gap: We come from this society, we go and study something new, and then we return to the same society and enter a world of dilemmas—starting with privacy and the therapy room: Who am I? Who are you? What counts as emotion? What counts as art? And we are a minority in a state where sometimes there is no sense of belonging or safety, which makes it even harder. At times you feel alone, and you realize you have to create something that fits—in language, in adaptation, in cultural sensitivity—everywhere you go.

Here, Moheba framed cultural adaptation not as a technical adjustment but as ongoing professional and existential labor. She described professional socialization as incomplete, requiring therapists to produce culturally viable knowledge and practices through lived experience, reflection, and further training. This narrative also situates embodiment and therapeutic work in minority positionality and local sociopolitical conditions that shape belonging, safety, and professional authority.

These accounts suggest that the gap between academic knowledge and clinical reality did not remain a point of disconnection but rather initiated a process of embodied inquiry and professional development. For many participants, they acquired knowledge through not only reading and lectures but also bodily practice, experiential learning, and lived experience. Miriam (46, clinical social worker) reflected:

I learned that as a therapist, I am supposed to continue my journey. For example, with your sexuality—to feel it, to be able to talk about such topics with clients. So, first I experience myself, believing that sexuality is a very basic issue in treatment. And I also enrolled in further focused training in this field.

Miriam’s account frames embodied knowledge not as optional enrichment, but as an ethical and professional necessity. Her narrative highlights a structural absence in academic training, in which central embodied dimensions of therapeutic work—such as sexuality and bodily presence—remain under-addressed. By seeking further training, she positioned herself as extending the boundaries of professional education, translating lived and embodied experience into clinical competence. In this sense, embodied learning becomes a means through which therapists challenge the limits of formal knowledge and reshape what counts as legitimate professional expertise.

Similarly, Jana (45, psychologist) pointed to a significant gap in her training: “The body is a very important tool in therapy, but in training it is almost absent. You learn concepts like transference and countertransference, but they don’t teach how they are expressed in the body.”

These accounts illustrate that academic studies do not end with the acquisition of formal knowledge but rather initiate a process of bodily and emotional internalization. For many participants, the professional journey became a personal journey in which they linked body, culture, and knowledge, and in doing so, they challenged the boundaries of professional training. In this context, participants voiced a clear call to expand academic discourse and training so that they include local knowledge, language, and practices from Arab culture. Ru’ya (39, dance and movement therapist) articulated this aspiration:

My goal is that when the Israeli Association of Creative and Expressive Therapies offers courses, they will give space to Arab culture as well. To bring it into academia. … It’s beginning to happen, even if in a small dose, but it is happening.

These therapists did not seek to dismantle the academic framework but to broaden it—to bring in cultural voices, local material, and the body as a locus of knowledge. This is a form of creative challenge rather than resistance: an expansion of what counts as legitimate therapeutic knowledge.

Participants described their commitment to this social mission. Beyond treating individuals, they said they see their work as contributing to the community and expanding professional and public awareness. As Tajreed (40, art and energy-healing therapist) put it: “This is not just therapy; it is a social mission. I don’t see myself only as a therapist, but also as someone who brings change—to break stereotypes, to show that things can be different.”

In this way, the therapists positioned themselves at the heart of broader processes of change. They used their knowledge, wisdom, and embodied experience as a bridge between emotion and action, between a distant academic language and the intimate reality of daily life. Their narratives show how the body has become a living, evolving, and meaningful source of knowledge—for them as women and professionals, for their clients, and for the communities they inhabit and seek to represent more fully in wider professional arenas.

## Discussion

The present study sought to understand how Arab Israeli women psychotherapists experience their bodies in therapeutic encounters and how this experience shapes their work. The findings indicate that the body functions as a central site through which knowledge is generated and emotional and relational processes unfold. Paying attention to the bodily experience (or listening to the body) emerged as a key mode of understanding therapeutic processes and mediating between Western psychological theories and local cultural practice. This approach echoes therapeutic traditions that emphasize the unity of body and mind (Freud, 1910; Lowen, 1979; Reich, 1933; Winnicott, 1965) while extending them to culturally specific contexts that have received limited attention in cross-cultural literature.

Whereas Western therapeutic models often emphasize individualism, emotional expression, and authenticity (Hofstede, 2001; Kitayama & Salvador, 2024), collectivistic settings such as Arab societies tend to prioritize social harmony, group norms, and the preservation of family honor (Abu-Baker, 2016). In these frameworks, women’s bodies are frequently subject to social monitoring and regulation, expressed through expectations regarding modesty, restraint, and visibility (Hasan, 2023). Benedict’s (1946) distinction between shame- and guilt-based cultures further highlights the role of the social gaze in shaping bodily and emotional expression. The findings suggest that therapists’ bodily experiences are deeply embedded in these cultural dynamics and cannot be understood apart from them.

Anthropological perspectives conceptualize the body as a primary site through which culture is lived and enacted (Blackman, 2021). In this sense, the bodily experiences described by Arab women therapists reflect ongoing negotiations between autonomy and restraint and between tradition and professionalization. The participants’ accounts demonstrate how the body operates as not only an emotional channel but also a locus of cultural mediation, where social norms, memories, and expectations are sensed and navigated in clinical practice.

These embodied negotiations are taking place against a backdrop of rapid social change in Arab society in Israel, including increased access to higher education, expanded professional roles for women, and growing exposure to psychological discourse (Haddad Haj-Yahya et al., 2022; Khamaisi, 2019; Nouman & Azaiza, 2023). Importantly, these transformations are not experienced solely at the level of beliefs or attitudes but are registered somatically—in breathing patterns, bodily tension, emotional expression, and professional boundaries. The findings suggest that bodily awareness is a key resource through which therapists manage the tensions generated by these intersecting social processes.

Arab women therapists described themselves as mediators who translate Western therapeutic concepts—such as authenticity, embodied awareness, and emotional attunement—into culturally sensitive practices aligned with norms of modesty and social caution. Through this work, they generate a hybrid therapeutic language that integrates academic knowledge with local cultural meanings (Asbeh & Nasra, 2014; Bar Zaken & Walsh, 2021). The body thus emerges as a central professional tool and cultural axis, enabling therapists to navigate between universal therapeutic ideals and the lived realities of their clients and communities.

At the clinical level, participants experienced their bodies as primary channels for understanding clients’ inner worlds. Through sensations, movement, breathing, and bodily affect, they accessed relational knowledge that was not always available through verbal communication alone. This aligns with theoretical perspectives emphasizing embodied awareness and the felt sense as routes to therapeutic understanding (Cornell, 2013; Totton, 2003). In the present study, however, bodily attunement also functioned as a culturally responsive strategy, offering an alternative pathway to emotional engagement when direct verbal expression was constrained.

Across participants’ narratives, embodied awareness emerged as an evolving process rather than a fixed professional competency. Although professional training often provided important opportunities to engage with bodily experience, participants also described becoming aware of their bodies through personal distress, supervision, clinical work, and other life experiences. Embodied awareness, therefore, was not understood as a capacity acquired solely through formal education, but as an ongoing process of personal and professional becoming. Through this process, the body gradually shifted from a site of restriction, surveillance, or invisibility to a source of knowledge, attunement, creativity, and professional agency. This transformation reflects broader sociocultural changes through which embodied awareness becomes intertwined with professional identity, ethical responsibility, and expanded feminine presence in traditional contexts.

Importantly, although several participants were trained in modalities that explicitly emphasize embodied processes, references to bodily awareness were not limited to therapists who used movement, art, or other experiential approaches. Psychologists, social workers, and family therapists likewise described attending to bodily sensations as a source of clinical understanding, emotional attunement, and professional reflection. This suggests that embodiment functioned as not only a modality-specific technique but also a broader dimension of therapeutic practice shaped by participants’ cultural, professional, and personal experiences. At the same time, differences in therapeutic orientation may influence how embodiment is conceptualized and integrated into clinical work, a question that warrants further investigation.

At the same time, participants frequently described tensions between knowledge acquired through professional training and the realities of clinical work in their communities. Accordingly, their embodied clinical work involved adapting and reshaping theoretical knowledge through lived experience, thereby challenging dominant assumptions about emotions, gender, and authority. In this sense, therapists acted as agents of social change in their communities, positioning the body as a site of dialog between tradition and innovation.

These findings resonate with contemporary literature on embodied and culturally mediated therapeutic processes (Buckley et al., 2018; White, 2018) while extending it by foregrounding the sociopolitical positioning of Arab women therapists in Israel. Embodiment, as described here, is not merely a personal or relational phenomenon, but one that is entangled with minority status, gendered surveillance, and ongoing negotiations of belonging and safety. The bodily processes articulated by participants thus reflect broader patterns of social change and professional transformation.

Rather than signifying a simple movement from cultural restriction to psychotherapy as a chance for liberation, participants’ accounts suggest an ongoing dialectic movement between different cultural and professional values. Ussher et al. (2017) described similar discourses, examining migrant and refugee women’s experiences of sexual embodiment and demonstrating how women negotiate dominant cultural discourses while maintaining agency and cultural belonging. Female therapists in the current study described developing embodied awareness through a continuous process of negotiating among professional knowledge, personal experience, and culturally embedded understandings of the body, including tensions surrounding touch, emotional expression, and the use of embodied therapeutic practices in religious and culturally conservative contexts. These findings suggest that embodiment is not merely a therapeutic resource but also can be seen as a sociocultural process through which cultural meanings, professional identities, and nonverbal forms of knowledge are continuously negotiated and transformed.

Taken together, the findings point to embodied awareness as a central component of personal, professional, and social expansion. Participants described a dynamic process in which learning to trust bodily sensations unfolded alongside shifts in self-permission, emotional openness, and cultural negotiation. Rather than abandoning cultural values, therapists reinterpreted them, creating space for embodied knowledge in therapeutic relationships. This movement—from a constrained body to a knowing, expressive body—emerged as a core dimension of professional identity and culturally responsive clinical practice.

### Limitations

This study explored the experiences of a specific group of Arab women psychotherapists working in the sociocultural context of Arab society in Israel. As with all qualitative research, the aim was not statistical representation but an in-depth exploration of meanings, processes, and embodied experiences in a particular context. Participants were drawn primarily from therapeutic approaches that involve embodied, expressive, or relational dimensions of practice, which may have heightened the salience of bodily awareness in their accounts. In addition, the sociopolitical context of Arab society in Israel may shape distinctive forms of bodily listening, professional identity, power dynamics, and therapeutic expectations that may manifest differently across varied cultural settings.

At the same time, the study represents a rich examination of how embodied experience is negotiated at the intersection of culture, gender, and professional practice. Rather than seeking population-level generalization, the findings provide conceptual insights into how bodily awareness functions in therapeutic work and may be relevant to other Arab, collectivistic, and traditionally oriented communities navigating similar tensions between body and psyche and between tradition norms and modern living as they manifest in various sociocultural settings.

### Practical Implications

At the practice level, the findings highlight the value of integrating nonverbal and embodied modalities, including art, movement, and other expressive approaches, in culturally responsive ways. Greater attention to culturally meaningful values, embodied practices, and locally grounded forms of knowledge may strengthen the accessibility, legitimacy, and relevance of psychotherapy in traditional communities and collectivistic cultural contexts.

Clinical supervision may similarly benefit from creating space for reflective dialog regarding psychotherapists’ bodily sensations, embodied countertransference, and the role of bodily awareness in therapeutic decision-making. Supporting therapists in exploring bodily experience as part of their professional development may enhance both clinical attunement and self-reflection. Moreover, our findings suggest that professional training programs may benefit from incorporating culturally adapted experiential opportunities to foster bodily awareness, sensory attunement, and reflection on embodied processes in therapeutic relationships. Such opportunities may support therapists in developing greater sensitivity to bodily experience as a source of clinical understanding while remaining attentive to cultural meanings and social norms.

## Conclusion

This study explored how Arab women therapists in Israel experience and make sense of bodily phenomena in psychotherapy. The findings suggest that embodied awareness is not merely a professional skill acquired through formal training, but also an ongoing process shaped through personal experience, clinical practice, cultural meanings, and professional development. Participants described the body as a source of knowledge, attunement, and therapeutic understanding while simultaneously negotiating cultural, gendered, and professional expectations. The study extends prior discussions of embodiment beyond individual psychological processes to highlight sociocultural dimensions through which bodily awareness is developed and expressed. The findings contribute to a culturally informed understanding of psychotherapy by suggesting that embodiment may serve as not only a therapeutic resource but also a site where personal histories, professional identities, and cultural meanings intersect and are continuously negotiated.

## Data Availability

No datasets were generated or analyzed during the current study.
